# Changes in Body Mass Index Following Pediatric Tonsillectomy for Obstructive Sleep Apnea

**DOI:** 10.7759/cureus.116877

**Published:** 2026-09-24

**Authors:** Hari J Roth, Bailey Balouch, Benjamin Rosen, Michael Nicolette, Logan Napoli, John Dibato, Donald Solomon

**Affiliations:** 1 Otolaryngology - Head and Neck Surgery, Cooper University Hospital, Camden, USA; 2 Internal Medicine, Rutgers New Jersey Medical School, Newark, USA; 3 General Surgery, Temple University Hospital, Philadelphia, USA; 4 Pediatrics, Nemours Children's Health System, Wilmington, USA; 5 Clinical Biostatistics, Cooper Medical School of Rowan University, Camden, USA

**Keywords:** body mass index, obstructive sleep apnea, pediatric tonsillectomy, surgical outcomes, tonsillectomy

## Abstract

Objective: The objective was to evaluate postoperative BMI trends (in kg/m²) in pediatric patients with obstructive sleep apnea (OSA) undergoing tonsillectomy by assessing BMI trends.

Study design: This study was a retrospective chart review.

Setting: This review took place at a single-institution tertiary care center.

Methods: Eight hundred four pediatric patients who underwent tonsillectomy at Cooper University Hospital between January 2020 and January 2022 were grouped by preprocedure diagnosis of OSA. Patient age, sex, BMI (kg/m²) at baseline and at one-, three-, six-, and 12-month follow-ups, and postoperative pain metrics were collected.

Results: For all 804 patients aged 0 to 17 years old, there were significant increases in BMI values at one, three, six, and 12 months postoperatively from a baseline of 21.2 kg/m². BMI increased at one month postoperatively (difference = 0.46921 kg/m^2^ (0.02183-0.9165), p = 0.0399), three months (difference = 0.6858 kg/m^2^ (0.1738-1.1978), p = 0.0088), six months (difference = 1.0025 kg/m^2^ (0.359-1.646), p = 0.0023), and 12 months (difference = 1.4458 kg/m^2^ (0.8234-2.0682), p < 0.0001). Stratified by age, 274 (34%) patients aged 0 to four years old with OSA had no significant changes from a baseline BMI of 17 kg/m² at one or three months postoperatively. BMI increased at six months (difference = 0.8626 kg/m^2^ (0.1929-1.5324), p = 0.012) and at 12 months (difference = 1.0068 kg/m^2^ (0.3047-1.7089), p = 0.0052). Three hundred five (38%) patients aged five to nine years old with OSA had no significant changes from a baseline BMI of 20.2 kg/m² at one or six months postoperatively. BMI increased at three months (difference = 1.185 kg/m^2^ (0.03143-2.3386), p = 0.0441) and at 12 months (difference = 1.0398 kg/m^2^ (0.3658-1.7138), p = 0.0027). Two hundred twenty-five (28%) patients aged 10 to 17 years old with OSA had no significant changes from a baseline BMI of 28.2 kg/m² at one, three, or six months postoperatively. BMI increased at 12 months (difference = 2.5252 kg/m^2^ (0.769-4.2813), p = 0.0053).

Conclusion: Tonsillectomy in pediatric patients with OSA is associated with significant postoperative BMI increases, highlighting the need for diagnosis-specific counseling and longitudinal weight monitoring.

## Introduction

Pediatric tonsillectomy is one of the most common surgical procedures performed in the United States. The most common indications include obstructive sleep apnea (OSA), recurrent tonsillitis, and peritonsillar abscess, among other less prevalent indications [1,2]. Approximately 500,000 children in the United States undergo tonsillectomy every year [3]. At present, an estimated 1-5% of American children have evidence of OSA or associated sleep-disordered breathing (SDB) [4]. 

Untreated pediatric OSA may lead to significant illness, morbidity, and health care utilization. It is associated with obesity, double the rate of respiratory tract infections, as well as potential cardiovascular disease, and behavioral symptoms such as fatigue and attention-deficit/hyperactivity disorder (ADHD)-like behavior [5]. Tonsillectomy remains a first-line surgical treatment for pediatric OSA and has been shown to improve overall quality of life and associated behavioral symptoms [6,7]. Surgical complications after tonsillectomy include postoperative bleeding, laryngospasm, laryngeal edema, and respiratory compromise [8] and are seen more frequently in patients with a diagnosis of OSA [9,10].

The literature offers mixed points of view of the effects of tonsillectomy on BMI. Postoperative weight fluctuation is likely multifactorial but discussion is vital in both preoperative and postoperative counselling [11]. One prior study demonstrated an improvement in obstructive symptoms with no change in BMI after tonsillectomy [12]. However, others demonstrated a significant increase in BMI during the postoperative period, with some associations with younger age [13] and prior BMI [14]. This has been both reinforced, and debated, in a systematic review [15], which denied an association between tonsillectomy and weight gain, but acknowledged that factors such as age and preoperative BMI could play a role in postoperative weight gain.

One hypothesized mechanism for possible weight loss following tonsillectomy for OSA includes fewer cortisol spikes during the night. This reduces overall cortisol levels and its associated effects on metabolism, something also done by continuous positive airway pressure (CPAP), providing a feasible physiologic mechanism for patients undergoing tonsillectomy for OSA to lose weight postoperatively [16,17]. However, contrasting studies hypothesized mechanisms for weight gain including decreased energy expenditure and decreased work of breathing during sleep [18].

Due to conflicting conclusions drawn by prior studies, as well as a wide range of endocrine and metabolic pathways that explain both weight gain and weight loss, it is evident that the effect of tonsillectomy on the postoperative weight of pediatric patients requires further investigation. This study primarily aimed to evaluate the impact of tonsillectomy on the weight of pediatric patients undergoing surgery for OSA at a single institution, as well as possible secondary outcomes, including postoperative pain metrics, analgesic requirements, and emergency department (ED) visits as these are common metrics and postoperative concerns when treating postoperative tonsillectomy.

## Materials and methods

This was a retrospective cohort study conducted at our tertiary care medical center, Cooper University Hospital, reviewing a database of tonsillectomy procedures performed between January 1, 2020, and January 1, 2022. Institutional Review Board approval was obtained before the initiation of the study (approval 22-153). A waiver of informed consent was obtained. All patients were under 18 years of age, underwent tonsillectomy, and had a pre-operative diagnosis of OSA based on either polysomnography or clinical assessment. Patients who did not have a preoperative diagnosis that included OSA were excluded from the study. Additionally, no distinction was made between patients who did or did not have formal polysomnography testing completed. Data collection included patient demographics, preoperative diagnosis, preoperative BMI, and postoperative BMI at one, three, six, and 12 months. Information was obtained by retrospective chart review, and BMI data was obtained by finding a charted value at any visit postoperatively. If BMI data was not obtainable for a time point, the patient did not have data entered for that time point, and statistical analysis was performed without this value. Variability in follow-up attendance and availability of BMI measurements at each postoperative time point may have introduced attrition bias. BMI values were included for one, three, six, and 12 months if the patients presented within two weeks of their one-month postoperative date, and within one month of their three-, six-, or 12-month postoperative date. Weight was measured using digital, in-office scales and height was obtained using manual measurements, and this was used to calculate BMI.

Patient demographics and baseline characteristics were analyzed using descriptive statistics and reported as the count and percentage or mean and standard deviation (SD). A Linear mixed model with the use of compound symmetry was used to assess changes in BMI values from baseline within the groups and different age categories to best assess postoperative change from preoperative BMI. The results from the model are represented as mean change (95% confidence interval (CI)). All statistical analysis was performed using SAS 9.4 (SAS Institute, Inc., Cary, NC) and p-values <0.05 were considered statistically significant.

## Results

Overall, 804 pediatric patients under the age of 18 who underwent tonsillectomy were included in our cohort, and all had a preoperative diagnosis of OSA. The average age was 7.1±3.9. There were 271 (35%) patients aged 0 to four years old, 293 (38%) aged five to nine, and 201 (26%) aged 10 to 17. There were 369 (48%) males and 396 females (52%) (Table 1). The average baseline BMI, BMI at one month, three months, six months, and 12 months were 21.2±6.7, 21.5±7, 22±6.5, 20.9±5.6, and 22.9±7.3, respectively. There were 692 patients (90%) who reported postoperative pain, 688 (90%) required postoperative pain medications, and 61 (8%) presented to the ED postoperatively (Table 1). Descriptive BMI values represent the unadjusted mean of available measurements at each postoperative time point, whereas the linear mixed model estimates changes from baseline while accounting for repeated measurements and unbalanced follow-up data. 

**Table 1:** Demographics of pediatric cohort undergoing tonsillectomy for cohort

Variables	N (%) / Mean (SD)
N	804 (100)
Age, yrs	7.1+/-3.9
Age groups	
0-4 yrs	274 (34)
5-9 yrs	305 (38)
10-17 yrs	225 (28)
Gender	
Male	369 (48)
Female	396 (52)
Baseline BMI	21.2+/-6.7 kg/m²
BMI at 1 month	21.5+/-7.0 kg/m²
BMI at 3 months	22.0+/-6.5 kg/m²
BMI at 6 months	20.9+/-5.6 kg/m²
BMI at 12 months	22.9+/-7.3 kg/m²
Post-op pain	692+/-90
Need for additional pain medication	688+/-90
ED visit for pain post-op	61+/-8

Mean change in BMI at one, three, six, and 12 months after tonsillectomy was analyzed using a linear mixed model with the use of compound symmetry to evaluate changes in BMI based upon age (Table 2). For all patients aged 0 to 17 years old there were significant increases in BMI at one, three, six, and 12 months postoperatively from a baseline of 21.2. BMI increased at one month postoperatively (difference = 0.46921 (0.02183-0.9165), p = 0.0399), three months (difference = 0.6858 (0.1738-1.1978), p = 0.0088), six months (difference = 1.0025 (0.359-1.646), p = 0.0023), and 12 months (difference = 1.4458 (0.8234-2.0682), p < 0.0001). When stratified by age, patients aged 0 to four years old with OSA had no significant changes from a baseline BMI of 17 at one or three months postoperatively. BMI increased at six months (difference = 0.8626 (0.1929-1.5324), p = 0.012) and at 12 months (difference = 1.0068 (0.3047-1.7089), p = 0.0052). Patients aged five to nine years old with OSA had no significant changes from a baseline BMI of 20.2 at one or six months postoperatively. BMI increased at three months (difference = 1.185 (0.03143-2.3386), p = 0.0441) and at 12 months (difference = 1.0398 (0.3658-1.7138), p = 0.0027). Patients aged 10 to 17 years old with OSA had no significant changes from a baseline BMI of 28.2 at one, three, or six months postoperatively. BMI increased at 12 months (difference = 2.5252 (0.769-4.2813), p = 0.0053).

**Table 2:** Linear mixed model demonstrating estimated change in BMI change from baseline BMI postoperatively A linear mixed model with the use of compound symmetry was used to assess changes in BMI values from baseline within the groups and different age categories. The results from the model are represented as mean change (95% confidence interval (CI)). All statistical analyses were performed using SAS 9.4 (SAS Institute, Inc., Cary, NC) and p-values <0.05 were considered statistically significant. LCI = lower confidence interval; UCI = upper confidence interval.

		Mean BMI change (kg/m^2 ^)	95% LCI	95% UCI	P value
Ages 0-17 years old (N=804)					
Baseline BMI	21.2				
BMI change at 1 month postoperatively		0.4692	0.02183	0.9165	0.0399
BMI change at 3 months postoperatively		0.6858	0.1738	1.1978	0.0088
BMI change at 6 months postoperatively		1.0025	0.359	1.646	0.0023
BMI change at 12 months postoperatively		1.4458	0.8234	2.0682	< .0001
0-4 years old (N=274)					
Baseline BMI	17.0				
BMI change at 1 month postoperatively		0.6264	-0.1276	1.3804	0.1028
BMI change at 3 months postoperatively		0.157	-0.2663	0.5804	0.4647
BMI change at 6 months postoperatively		0.8626	0.1929	1.5324	0.012
BMI at 12 months postoperatively		1.0068	0.3047	1.7089	0.0052
5-9 yrs years old (N=305)					
Baseline BMI postoperatively	20.2				
BMI change at 1 month postoperatively		0.4784	-0.2324	1.1892	0.1858
BMI change at 3 months postoperatively		1.185	0.03143	2.3386	0.0441
BMI change at 6 months postoperatively		0.6367	-0.3539	1.6274	0.2063
BMI change at 12 months postoperatively		1.0398	0.3658	1.7138	0.0027
10-17 years old (N=225)					
Baseline BMI	28.2				
BMI change at 1 month postoperatively		0.2127	-0.6376	1.0629	0.6208
BMI change at 3 months postoperatively		0.6472	-0.1497	1.444	0.1103
BMI change at 6 months postoperatively		1.9024	-0.0886	3.8934	0.0609
BMI change at 12 months postoperatively		2.5252	0.769	4.2813	0.0053

## Discussion

The results of this study provide valuable insight into the impact of tonsillectomy on the BMI of pediatric patients. In our cohort, patients demonstrated a significant increase in BMI at 12 months postoperatively regardless of age, and when controlling for age, demonstrated significant increases at one, three, or six months postoperatively depending upon age at time of surgery. Mechanisms for these findings have not been fully characterized, although several hypotheses exist for why improvement in OSA might trigger an increase in BMI. Cessation of SDB can result in children spending less energy on nocturnal breathing and therefore energy expenditure, resulting in a consequent increase in BMI [18]. Other studies have reiterated this theory [19,20], and also demonstrated that children with failure-to-thrive experience more significant increases in BMI after tonsillectomy. Further mechanisms have been described in a twin study, which demonstrated OSA requiring tonsillectomy was associated with lower baseline levels of insulin growth factor-1 (IGF-1) three months postoperatively [21]. Studies show that treatment of OSA via CPAP decreased cortisol levels [16,17] and its presence is known to increase insulin resistance and blood glucose levels [16,17,22]. Additionally, untreated OSA is associated with elevated sympathetic tone in adults [23,24] and children [25] and, therefore, energy expenditure; consequently, its relief might tilt energy balance towards storage. These proposed metabolic and endocrine mechanisms remain hypothetical in the context of our study, as these pathways were not directly evaluated in our cohort.

The literature on clinical outcomes in post-tonsillectomy pediatric patients largely mirrors that of the biochemical literature in that there is evidence both for and against weight gain after tonsillectomy, and there are many rising theories as to why this may be the case. Tonsillectomy has been shown to be associated with weight gain regardless of surgical indication in prior retrospective cohort studies, but this remained independent of surgical indication and was more significant in ages under six years old [20]. Similar findings have been reported, with emphasis on early age at time of operation [26]. Our cohort differs in this regard, as we reported older age being associated with more significant changes in BMI postoperatively. This could potentially be accounted for by differences in severity of OSA. In children with mild OSA, there was found to be no significant weight gain after surgery [27] compared with conservative management. As OSA remains untreated and progresses with age, it is possible that postoperative weight gain is associated with disease severity and baseline growth.

Our findings contribute to the expanding literature on tonsillectomy and pediatric BMI, and as a result can assist in guiding preoperative counselling and postoperative management of patients undergoing tonsillectomy. Patients and families should be warned about the risk of weight gain postoperatively, and have accurate weight checks during follow-up visits. Likewise, early referral to dietitians or obesity-management programs should be considered if rapid weight gain is observed [28]. The effects of tonsillectomy expand far beyond the realm of Otolaryngology due to the effects on BMI demonstrated. Childhood obesity is a growing epidemic with profound relevance to pediatric OSA. In the United States, nearly one in five children and adolescents are obese [29], with some studies citing up to 50-60% of these children having OSA [30]. The immense health impacts of obesity are well documented and described in the medical community [31], and as a result tonsillectomy provides a valuable opportunity to counsel on risk factors and reduce incidence at a young age. Interventions such as family healthy weight programs (FHWPs) endorsed by the American Academy of Pediatrics can serve as invaluable resources for patients undergoing tonsillectomy, especially those that are identified to be at increased risk for postoperative weight gain. FHWPs such as the Mind, Exercise, Nutrition, Do it (MEND) Program have been shown to have positive impacts on BMI [32] through multidisciplinary teams focusing on actionable goals such as nutrition, physical activity and behavioral counseling with the help of family, primary care providers, nutritionists, obesity specialists, and therapists.

Higher BMI and older age are strongly associated with increased and more severe postoperative pain immediately after surgery [33]. Current American Academy of Otolaryngology-Head and Neck Surgery (AAO-HNS) guidelines recommend a single intraoperative dose of dexamethasone and routine postoperative acetaminophen and ibuprofen for all children undergoing tonsillectomy. Guidelines from the AAO-HNS also recommend overnight inpatient stays for any patients under three or those with severe OSA (defined as apnea-hypopnea index (AHI) over 10 or with significant desaturations) [6]. Adherence to these recommendations is especially important in high-risk patients like those with OSA, and especially those who are obese and have increased concern for respiratory depression. In an effort to balance these concerns and risks, our findings demonstrate the importance of identifying patients at high risk for postoperative pain, such as the obese population undergoing tonsillectomy. In these cases, patients may require extended courses of analgesics and closer follow-up. Families should receive clear instructions on dosing schedules and on indicators for medical evaluation such as respiratory depression, dehydration, and inadequate pain relief.

Our study was limited by its retrospective nature. Likewise, retrospective chart review for BMI values after 12 months proved difficult, and further prospective studies would benefit from further trending BMI for periods longer than 12 months postoperatively. Although the increase in BMI seen after tonsillectomy was statistically significant, it is unclear whether the increase was clinically significant, particularly given that absolute BMI varies with age and sex during normal pediatric growth. Without the use of age- and sex-specific BMI percentiles or z-scores, these changes cannot be definitively distinguished from normal physiological growth or interpreted as increased adiposity. Additionally, the absence of a nonsurgical control group limits our ability to distinguish postoperative BMI changes associated with tonsillectomy from those expected during normal childhood growth and development. Further elucidation of the clinical significance of the observed BMI change would require long-term follow-up with additional health outcome measures as well as patient-centered outcomes focused on impact of BMI change on quality of life. Also, patients with OSA were included whether or not they had undergone formal polysomnography testing. This decision was made largely as a result of variability in practice of pre-operative testing and physician testing preference given patients’ unique circumstances and clinical presentation. A diagnosis of OSA was based upon clinical assessment with or without formal polysomnography testing, which therefore includes patients with SDB in the OSA group.

Additionally, further research should explore the endocrine and metabolic pathways linking OSA to growth changes. Further prospective studies measuring hormone levels such as IGF-1 and other related hormones such as leptin and ghrelin before and after tonsillectomy to clarify mechanisms of phenomena seen in the literature. Also, longer-term follow-up is needed to determine whether early BMI increase persists, plateaus, or declines in later adolescence or in early adulthood. Likewise, comparison to natural BMI growth curves published by the CDC could prove useful in cases where control subjects could not be studied. BMI curves are individualized for age and gender, and studies such as this will require future large-scale analysis to match subjects to their controls. Generally, BMI peaks around one year of age with a decline shortly after until age five or six years of age, where it then gradually increases to ages 12 to 14 years old [34,35]. Understanding these patterns could inform targeted interventions to optimize obesity prevention and guide postoperative planning and management of this large population of patients.

## Conclusions

This study examined the impact of tonsillectomy on BMI in pediatric patients, revealing a statistically significant increase in BMI at 12 months, with similar trends observed at three and six months, warranting further investigation through larger or prospective studies to further assess this association. These findings contribute to the evolving understanding of the relationship between tonsillectomy and BMI, highlighting the importance of nuanced, evidence-based postoperative counseling and addressing pediatric obesity risks.
